# The Timing of Kinematic and Kinetic Parameters during Gait Cycle as a Marker of Early Gait Deterioration in Multiple Sclerosis Subjects with Mild Disability

**DOI:** 10.3390/jcm11071892

**Published:** 2022-03-29

**Authors:** Francisco Molina-Rueda, Diego Fernández-Vázquez, Víctor Navarro-López, Juan Carlos Miangolarra-Page, María Carratalá-Tejada

**Affiliations:** 1 Physical Therapy, Occupational Therapy, Rehabilitation and Physical Medicine Department, Faculty of Health Sciences, Rey Juan Carlos University, 28922 Madrid, Spain; francisco.molina@urjc.es (F.M.-R.); victor.navarro@urjc.es (V.N.-L.); juan.miangolarra@urjc.es (J.C.M.-P.); maria.carratala@urjc.es (M.C.-T.); 2 Movement Analysis, Biomechanics, Ergonomics, and Motor Control Laboratory, Faculty of Health Sciences, Rey Juan Carlos University, 28922 Madrid, Spain; 3 Physical Medicine and Rehabilitation Service, University Hospital of Fuenlabrada, 28942 Madrid, Spain

**Keywords:** gait analysis, multiple sclerosis, biomechanical phenomena

## Abstract

This study aimed to evaluate walking in multiple sclerosis (MS) patients with mild disability. A case control study with 8 mild disability MS patients and 10 controls was conducted. This study analyzed spatiotemporal, kinematic, and kinetic parameters. We also analyzed the timing of these parameters, as a percentage of the gait cycle. The MS patients and controls walked with a similar gait pattern. However, there were differences in the timing of the biomechanical parameters. The timing of toe-off was at 62–63% of gait cycle in MS subjects while in controls it was at 59.94% (*p* = 0.009 to 0.027 vs. to controls). The peak of knee flexion during swing was at 74–76% of gait cycle in MS subjects while in controls was at 72% (*p* = 0.027 to 0.034). While the peak of ankle dorsiflexion during stance occurred at 48–50% in MS subjects, while in controls it was at 46% (*p* = 0.001 to 0.009), and the peak of plantar flexion during pre-swing was at 66% in MS subjects vs. 64% in controls (*p* = 0.001). At the kinetic pattern, the first peak of the vertical ground reaction force occurred at 14% of gait cycle in controls while in MS patients it was at 17–20% (*p* = 0.012 to 0.021). MS subjects with mild disability walked with similar spatiotemporal parameters, joint angles and moments compared to controls. However, our results suggest that those changed the temporal occurrences, expressed as percentage of the gait cycle, of the kinematic and kinetic parameters.

## 1. Introduction

People with multiple sclerosis (MS) usually present a relentless, progressive decline of neurologic functionating, commonly affecting locomotion, bladder function, and cognition [1,2]. There is evidence for gait abnormalities in subjects with MS who have mild disability compared with healthy subjects using three-dimensional motion capture systems [3]. Specifically, these subjects walked with a reduced speed, stride length, and cadence compared with the controls [3]. These alterations are associated with a higher risk of falls in these patients [4]. In addition, different authors have reported that the MS patients with mild disability demonstrated kinematic asymmetries in the sagittal plane. They observed a reduced hip extension and hip range of motion during the stance period [5]. These findings are more remarkable in the MS patients who have spastic signs [6]. At the ankle joint, several authors have described reduced ankle dorsiflexion during the initial contact and a reduced plantarflexion in the pre-swing phase [7]. By comparison, such impairments have not been captured clinically by non-instrumented performance-based tests [3,8].

Biomechanical parameters of gait can be obtained using three-dimensional motion capture systems. To date, these systems have registered gait spatiotemporal (velocity, cadence, stride length) kinematics and kinetics in the MS subjects who have mild disability (i.e., Expanded Disability Status Scale (EDSS) scores ≤ 3.5) [9]) but have not focused on temporal parameters. Temporal parameters correspond to temporal occurrences, expressed as the percentage of the gait cycle, and to the kinematic and kinetic parameters. For example, the peaks of the hip extension and knee flexion during normal walking occur approximately at 50% and 70% of the gait cycle completion, respectively [10,11]. These percentages may change with the variability of speed or double support period during walking [12]. Specifically, a change in double support percentage affects the kinematic parameters and the percentage of the gait cycle in which these parameters occur [13]. Therefore, it is possible that the MS patients with mild disability modify the timing of several kinematic and kinetic parameters.

Consequently, an attempt to obtain the timing of kinematic and kinetic parameters during the gait cycle in the MS patients with mild disability may offer valuable information about their gait impairment. Earlier detection of gait alterations in subjects with MS is essential because it could identify targets of rehabilitation at an earlier disease course.

This observational case-control study aimed to evaluate the kinematic and kinetic pattern (angles, moments, ground reaction forces, and timing) during walking in the MS patients with mild disability.

## 2. Materials and Methods

### 2.1. Participants

Voluntary participation was solicited from the subjects with MS, with recruitment beginning in September 2020. The patients had to meet the following inclusion criteria: aged over 18 years old; diagnosed with MS based on the McDonald criteria [14]; EDSS score less than or equal to 3.5 [15]. We excluded participants who over the previous six months had suffered a worsening of symptoms; had required hospitalization; corticoid therapy, either intravenous or oral; botulinum toxin; or experienced any other situation that could potentially hamper their participation in the study. The inclusion criteria for the control subjects included walking independently without assistive devices and the absence of musculoskeletal and/or neurological disorders.

The Research Ethics Committee of the Local University approved the study and informed consent was obtained from all participants.

### 2.2. Experimental Protocol

The experimental protocol began in November 2020 and was performed according to the STROBE checklist.

The instrumental analysis of the gait was recorded by Vicon Motion System^®^ (Vicon Motion Systems, Oxford, UK) using 8 MX 13+ infrared capture cameras and three AMTI^®^ (Accent Micro Technologies Inc., Watertown, MA, USA) dynamometric force platforms (410 × 610 mm), located in the middle of an 11 m walking corridor. Special lightweight surface markers were attached directly to the skin and placed over standardized landmarks on the pelvis and both limbs (anterior and posterior superior iliac spines; lateral thigh; lateral femoral condyle; lateral leg; lateral malleoli; second metatarsal distal head; and the posterior heel) according to the biomechanical models of Kadaba et al. [16] and Davis et al. [17]. After instrumentation, the subjects were asked to walk at a self-selected comfortable gait speed by corridor lab, recording five repetitions per subject in each session. A successful test was one in which both feet landed fully on, at least, two of the three force platforms, one foot on each platform. The Vicon^®^ Nexus software v1.8.5 was used to calculate outcome measures based on the biomechanical model of the Vicon^®^ Plug-in Gait. To avoid possible bias, the evaluator who performed the processing of the gait tests was independent of the researcher in charge of the analysis of the results. The same protocol was followed for both study groups.

### 2.3. Outcomes Measures

The spatiotemporal parameters analyzed in this study were stride length, velocity, cadence, and timing of the toe-off (expressed as percentage of the gait cycle). The following kinematic parameters were analyzed: pelvis range of motion (ROM) in the sagittal; hip ROM; hip angle at initial contact; peak hip extension during stance period; peak hip flexion during swing period; knee ROM; knee angle at initial contact; peak knee flexion in loading response; peak knee flexion during swing period; ankle ROM; ankle at initial contact; peak ankle dorsiflexion in stance period; and peak ankle plantarflexion at the toe-off. We also analyzed the timing of the kinematic parameters, expressed as a percentage of the gait cycle.

Regarding the kinetic parameters, in this study we analyzed the joint internal moments and the vertical ground reaction force (GRF). The following kinetic parameters were analyzed: the peak hip extensor moment; the peak knee extensor moment during loading response phase; the peak ankle plantar flexor moment during the pre-swing phase; and the first and second peaks of the vertical GRF. We also analyzed the percentage of the gait cycle in which these kinetics events occurred.

Both legs were analyzed in people with multiple sclerosis, which we classified as more affected lower extremity (LE) and less affected LE. In the controls, the dominant lower extremity was analyzed. An average of five motion capture recordings was made for each lower extremity.

### 2.4. Data Analysis

The output angles for all joints were calculated from the YXZ cardinal angles derived by comparing the relative orientations of the two segments, safe pelvis, that is an absolute value, and were measured relative to the laboratory axes [18]. The position of the hip segment was relative to the proximal segment, i.e., the hip to the pelvis. The course and direction of the segment axes are shown in the Vicon^®^ Plug-in Gait Product Guide-Foundation Notes Revision [19]. Joint internal moment calculations were determined from synchronized coordinates and force data using an inverse dynamics approach [20]. Joint kinetics were normalized to body weight, and all parameters were normalized to 100% of the gait cycle.

Statistical analysis was performed using SPSS 27.0, and Shapiro and Wilk’s W-statistic was used to screen all data for normality of distribution. Descriptive statistics were used to analyze quantitative data (median ± interquartile range). The U-Mann–Whitney test was used to determine whether significant differences existed between groups. A statistical threshold of *p* ≤ 0.05 was considered significant.

Effect size was calculated using the Cohen’s *d*, 0.2 was considered small, 0.5 medium, and 0.8 large.

## 3. Results

A total of 8 MS patients and 10 healthy controls were included in this study (Table 1). The median and interquartile range of the EDSS of the MS subjects was 2.5 (2.6) (EDSS between 0 and 3). No patients had a diagnosis of the progressive type of MS.

According to the spatiotemporal parameters (Table 2), the MS patients walked with similar stride length, velocity, and cadence to the non-disabled subjects. The timing of toe-off was at 59.94% of gait cycle in the controls, while in the MS subjects was at 63.22% (*p* = 0.027; Cohen’s *d* = 0.523) in more affected LE and 62.81% (*p* = 0.009; Cohen’s *d* = 0.607) in less affected LE.

There were no differences between groups in pelvis, hip, knee, and angle degrees during walking. However, the statistical analyses showed significant differences in the temporal parameters. At the knee joint, the peak of flexion during swing was at 74% (*p* = 0.027; Cohen’s *d* = 0.541) in the more affected LE and 76% (*p* = 0.034; Cohen’s *d* = 0.519) in less affected LE of gait cycle in the MS subjects while in the controls was at 72%. At the ankle joint, the peak of dorsiflexion during stance occurs at 50% (*p* = 0.001; Cohen’s *d* = 0.766) in more affected LE and 48% (*p* = 0.009; Cohen’s *d* = 0.627) in less affected LE of gait cycle in the MS subjects while in the controls was at 46%. In addition, the peak of plantarflexion during pre-swing was at 66% (both lower limbs) (more affected LE *p* = 0.001; Cohen’s *d* = 0.755; less affected LE *p* = 0.001; Cohen’s *d* = 0.749) of gait cycle in the MS subjects vs. 64% in the controls. Data are summarized as a median and interquartile range in Table 3.

At the kinetic pattern, there were no statistical differences between groups in the magnitude of joint moments and vertical ground reaction forces. However, we registered significant differences in the percentages of the gait cycle in which the peak values occur. The main finding was observed in the first peak of the vertical ground reaction force. This parameter occurs at 14% of gait cycle in the controls while in the MS patients it was at 20% (*p* = 0.021 Cohen’s *d* = 0.564) in more affected LE and 17% (*p* = 0.012; Cohen’s *d* = 0.611) in less affected LE. Data are expressed as a median and interquartile range in Table 4.

## 4. Discussion

In this observational study, alterations in gait parameters were identified in the MS subjects with mild disability compared to the healthy controls.

We observed that the MS subjects with mild disability walked with similar joint angles and moments compared to controls. In addition, we did not register changes in the spatiotemporal parameters.

**Figure 1:**
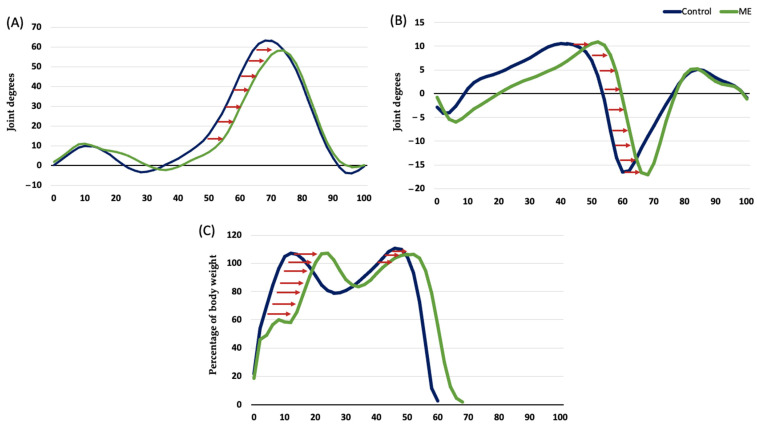
(**A**) Timing of the knee kinematics; (**B**) Timing of the ankle kinematics; (**C**) Timing of the vertical ground reaction forces. (**A**–**C**) *x*-axis is percentage of the gait cycle. (**A**,**B**) *y*-axis is joint degrees. (**C**) *y*-axis is percentage of body weight. The blue line is the control group; the green line is the MS group.

Regarding the temporal parameters, we observed tendencies mostly related to the lengthening of the stance phase (delayed toe-off). Specifically, we showed that the peak of the knee flexion during swing was at 74–76% of the gait cycle in comparison with the 72% observed in the controls. The same tendency was observed for the timing of ankle dorsiflexion (50% for more affected LE and 48% for less affected LE vs. 46% in the controls) and plantar flexion (66% for both lower limbs vs. 64% in the controls) (Figure 1). In relation to the kinetic parameters, the MS subjects with mild disability showed changes in the timing of some parameters at the more affected lower limb: knee extension moment on stance, ankle plantarflexion on pre-swing, and both peaks of the vertical ground reaction forces (Figure 1). For most of the significant findings, a medium effect size was observed.

According to Benedetti et al. [5], the gait pattern among the MS subjects with mild disability was characterized by an increased hip flexion and knee flexion at initial contact followed by a reduced hip extension [6,21] and ankle plantarflexion in the stance period compared to the non-disabled controls. During the swing period, an increased hip flexion was reported. On the other hand, Sosnoff et al. [3] reported that the MS subjects with minimal disability have subtle but detectable differences in gait spatiotemporal parameters with clinically feasible technology. In addition, Huisinga et al. [21] registered alterations in the joint moments in people with MS during walking. They showed a decrease in the magnitude of several joint moments: ankle dorsiflexor and knee extensor during early stance and ankle plantar flexor and hip flexor during late stance. These results were not observed in our study.

Surprisingly, our results indicate a symmetrical gait pattern in MS people with mild disability without clinical evidence of abnormal walking and without clinical evidence of muscle alteration or increased muscle tone as measured by a passive range of motion. One possible reason that could explain the differences between our work and the previous research is the functional level of the participants included. Benedetti et al. [5] recruited into their study some subjects with minimal functional involvement of walking steadiness and smoothness. Sosnoff et al. [3] included MS participants with EDSS that ranged from 1 to 3.5 and Huisinga et al. [21] between 1 and 4. In our study, the patients were all independent walkers with EDSS scores ≤ 3 (ranged from 0 to 3).

Contrary to what was reported by Benedetti et al. [5] on the minimally impaired patients, Severini et al. [22] did not observe significant differences between the controls and the MS subjects with mild disability for any of the hip and knee parameters. In agreement with our results, they observed some differences in the timing of kinematic parameters. Specifically, the analysis revealed differences in the timing of maximal dorsiflexion during swing. For the EDSS-based stratification, Severini et al. [22] observed differences between the MS patients with mild disability and the controls only at the ankle level (reduced peak plantar flexion during the stance period). This difference in results is possibly related to muscle weakness and spasticity, given the difference in EDSS values of the patients between our sample (EDSS ≤ 3) and that examined in Severini et al., 2017 (EDSS < 5). In this sense, several studies have described significant reductions in maximal ankle plantar flexion in the MS subjects with EDSS scores ranging from 0 to 4 [5,21,23] and increased muscle tone.

Although there is no consensus in the previous literature on gait disturbances in people with MS with mild disability, gait analysis provides evidence of minimal dysfunction at a level not yet perceptible by a standard examination (subclinical dysfunction). Therefore, the information obtained from three-dimensional motion capture systems could be considered to prevent future alterations in the gait pattern of people with MS.

The change in the timing of the kinematic and kinetic parameters and the lengthening of the stance phase could be early markers of muscle weakness or the beginning of altered muscle tone. In fact, the different temporal parameters observed in this study correspond to the deficit in kinematic and kinetic events described in the MS subjects with moderate or severe disability: decreased dorsiflexion during the stance; restricted plantar flexion during the toe-off; reduced knee flexion during the swing period [24]; and decreased knee extension moment on stance and ankle plantar flexion on pre-swing [21]. Therefore, this finding has clinical relevance, as it could guide health professionals in their gait observation of those subjects with EDSS ≤ 3.

## 5. Study Limitations

There are several limitations in this study. Because our sample of subjects with MS was rather small, caution must be observed in generalizing the results. Therefore, future studies with a larger sample and with comparable groups in terms of sex and age are necessary, since they are variables that influence the gait pattern [25]. Our study did not include any electromyography lower extremity data, which limits the interpretation of our results. Another limitation is the variability of the data. In our study, the standard deviation was very high for some parameters.

## 6. Conclusions

The MS subjects with mild disability (EDSS ≤ 3) walked with similar spatiotemporal parameters, joint angles and moments compared to the controls. However, our results suggest that MS patients with mild disability change, delaying the temporal occurrences expressed as percentage of the gait cycle, the kinematic and kinetic parameters. These findings may favor an early gait rehabilitation in MS subjects with mild disability.

## Figures and Tables

**Table 1:** Demographic information for MS patients and healthy controls.

Parameters	MS (*n* = 8)	Healthy Controls (*n* = 10)
Sex	6 female, 2 male	7 female, 3 male
Age	35 ± 8.23	31 ± 6
Height (cm)	171.25 ± 7.67	170.45 ± 8.22
Mass (Kg)	71.35 ± 7.01	63.44 ± 9.89
Years Since Diagnosis	8.25 ± 6.32	-

**Table 2:** Spatiotemporal parameters.

Parameters	Median (IR)			MALE vs. DCL		LALE vs. DCL		MALE vs. LALE	
	MALE	LALE	DCL	*p*	*d*	*p*	*d*	*p*	*d*
Stride length (m)	1.25 (0.21)	1.25 (0.21)	1.28 (0.17)	0.173	0.335	0.146	0.356	0.798	0.062
Velocity (m/s)	1.25 (0.33)	1.25 (0.33)	1.3 (0.11)	0.068	0.43	0.068	0.43	1	0
Cadence (steps)	111 (18)	111 (18)	118.91 (13.86)	0.460	0.189	0.460	0.189	0.959	0.025
Percentage of toe-off (%)	63.22 (4)	62.81 (5)	59.94 (2)	0.027	0.523	0.009	0.607	0.878	0.049

Subsubsection MALE: More Affected Lower Extremity; LALE: Less Affected Lower Extremity; DCL: Dominant Control Limb; IR Interquartile Range; *p* < 0.05; *d*: Cohen’s *d*.

**Table 3:** Kinematic Data.

Parameters	Median (IR)			MALE vs. DCL		LALE vs. DCL		MALE vs. LALE	
	MALE	LALE	DCL	*p*	*d*	*p*	*d*	*p*	*d*
Pelvis ROM sagittal plane (°)	3.03 (2.26)	2.82 (2.09)	2.22 (0.67)	0.122	0.377	0.122	0.377	0.959	0.025
Hip ROM sagittal plane(°)	41.15 (7.82)	43.44 (10.47)	42 (5.03)	0.965	0.021	1	0	0.959	0.025
Hip angle at IC (°)	30.82 (1.97)	30.55 (8.38)	29.6 (8.87)	0.475	0.184	0.887	0.046	0.805	0.075
Peak hip extension during stance period (°)	−9.8 (4.54)	−11.39 (5.28)	−11.12 (10.27)	0.887	0.046	0.887	0.046	0.318	0.256
Timing of peak hip extension during stance period (%)	54 (6)	54 (2)	52 (3)	0.270	0.276	0.070	0.457	0.902	0.031
Peak hip flexion during swing period (°)	32.39 (3.89)	30.71 (9.35)	31.84 (9.49)	0.887	0.046	1	0	0.805	0.075
Timing Peak hip flexion during swing period (%)	98 (6)	100 (12)	91 (12)	0.315	0.259	0.109	0.392	0.535	0.173
Knee ROM (°)	58.84 (6.65)	59.5 (11.1)	59.07 (9.01)	0.762	0.084	0.897	0.042	0.574	0.148
Knee angle at IC (°)	3.32 (4.26)	4.68 (5.33)	2.5 (4.62)	0.408	0.21	0.573	0.147	0.959	0.025
Peak knee flexion in load response (°)	11.19 (9.02)	10.68 (7.73)	10.98 (5.9)	0.965	0.021	1	0	0.798	0.074
Timing of Peak knee flexion in load response (%)	12 (2)	12 (2)	12 (2)	0.633	0.141	0.515	0.18	0.442	0.195
Peak knee flexion during swing period (°)	56.63 (10.11)	58.98 (11.53)	58.08 (9.86)	0.633	0.126	0.460	0.189	0.878	0.049
Timing Peak knee flexion during swing period (%)	74 (2)	76 (2)	72 (3)	0.027	0.541	0.034	0.519	0.574	0.153
Ankle ROM sagittal plane (°)	30.64 (7.71)	27.97 (7.68)	31.69 (6.69)	0.315	0.251	0.203	0.314	0.959	0.025
Ankle at IC (°)	−2.58 (4.8)	−0.32 (5.48)	−0.23 (4.49)	0.696	0.105	0.633	0.126	0.328	0.247
Peak ankle dorsiflexion in stance period (°)	16.85 (6.94)	16.46 (6.16)	16.68 (9.2)	1	0	0.965	0.021	0.721	0.099
Timing of peak ankle dorsiflexion in stance period (%)	50 (4)	48 (6)	46 (3)	0.001	0.766	0.009	0.627	0.505	0.182
Peak ankle plantarflexion at the toe-off (°)	−14.83 (12.74)	−16.72 (9.16)	−14.45 (8.88)	0.696	0.105	0.897	0.042	0.798	0.074
Timing of peak ankle plantarflexion at the toe-off (%)	66 (2)	66 (4)	64 (2)	0.001	0.755	0.001	0.749	0.505	0.174

MALE: More Affected Lower Extremity; LALE: Less Affected Lower Extremity; DCL: Dominant Control Lower Limb; IR Interquartile Range; ROM Range of Motion; *p* < 0.05; *d*: Cohen’s *d*.

**Table 4:** Kinetic data.

Parameters	Median (IR)			MALE vs. DCL		LALE vs. DCL		MALE vs. LALE	
	MALE	LALE	DCL	*p*	*d*	*p*	*d*	*p*	*d*
Peak hip extensor moment (Nm/kg)	0.7 (0.71)	0.59 (0.62)	0.85 (0.24)	0.515	0.168	0.315	0.251	0.959	0.025
Timing of peak hip extensor moment (%)	4 (4)	0 (4)	3 (4)	0.829	0.056	0.315	0.26	0.328	0.264
Peak knee extensor moment during loading response phase (Nm/kg)	0.4 (0.45)	0.3 (0.56)	0.4 (0.35)	0.696	0.105	0.573	0.147	0.878	0.049
Timing of peak knee extensor moment during loading response phase (%)	14 (9)	14 (2)	12 (2)	0.003	0.688	0.068	0.447	0.161	0.384
Peak ankle plantar flexor moment during pre-swing phase (Nm/kg)	1.41 (0.44)	1.47 (0.4)	1.54 (0.29)	0.173	0.335	0.633	0.126	0.382	0.223
Timing of peak ankle plantar flexor moment during pre-swing phase (%)	50 (3)	50 (4)	48 (1)	0.034	0.564	0.101	0.459	0.798	0.079
First peak of the Vertical Ground Reaction Force (Nm/kg)	104.07 (15.58)	106.7 (12.08)	104.5 (5.67)	0.829	0.063	0.965	0.021	1	0
Timing of the first peak of the Vertical Ground Reaction Force (%)	20 (6)	17 (4)	14 (3)	0.021	0.564	0.012	0.611	0.645	0.114
Second peak of the Vertical Ground Reaction Force (Nm/kg)	112.07 (10.39)	112.7 (12.8)	115.54 (9.71)	0.360	0.231	0.633	0.126	0.574	0.148
Timing of the second peak Vertical Ground Reaction Force (%)	49 (2)	48 (4)	48 (2)	0.043	0.522	0.083	0.466	0.959	0.026

MALE: More Affected Lower Extremity; LALE: Less Affected Lower Extremity; DCL: Dominant Control Lower Limb; IR Interquartile Range; *p* < 0.05; *d*: Cohen’s *d*.
